# The relationship between spinal pain and temporomandibular joint disorders in Korea: a nationwide propensity score-matched study

**DOI:** 10.1186/s12891-019-3003-4

**Published:** 2019-12-29

**Authors:** Doori Kim, Seong-Gyu Ko, Eun-Kyoung Lee, Boyoung Jung

**Affiliations:** 1 Jaseng Spine and Joint Research Institute, Jaseng Medical Foundation, 3F, 538 Gangnam-daero, Gangnam-gu, Seoul, 06110 Republic of Korea; 2 Department of Preventive Medicine, College of Korean Medicine, Graduate School, Khyung Hee University, Hoegi-dong, Dongdaemun-gu, Seoul, 02453 Republic of Korea; 3 Research Department, Research Institute of Korean Medicine Policy, 91, Heojun-ro, Gangseo-gu, Seoul, 07525 Republic of Korea

**Keywords:** Back pain, Health insurance review and assessment National Patients Sample (HIRA-NPS), Medical service use, Musculoskeletal pain, Propensity score matching, Temporomandibular joint pain

## Abstract

**Background:**

Patients with temporomandibular joint disorder (TMD) often complain of pain in other areas. Several studies have been conducted on spinal pain in TMD patients, but have contained only limited information. Therefore, this study analyzed the relationship between TMD and spinal pain in greater detail by using nationwide data.

**Methods:**

A total of 12,375 TMD patients from the Korean National Health Insurance Review and Assessment database were analyzed. Controls were selected using propensity score-matching. The McNemar test, chi-square test, and paired t-test were used to compare the prevalence and severity of spinal pain between cases and matched controls. Logistic regression and linear regression models were used to analyze factors affecting the prevalence and severity of spinal pain in patients with TMD.

**Results:**

The annual period prevalence of TMD was 1.1%. The prevalence was higher in younger individuals than in individuals of other ages and was higher in women than in men. The medical expenditure for TMD per person was $86. Among TMD patients, 2.5% underwent surgical procedures and 0.3% were hospitalized. The prevalence of spinal pain in patients with TMD was 48%, whereas that in the control group was 34%. Increased severity of TMD was associated with an increased probability of spinal pain. The medical expenditure, mean number of visits, and lengths of treatment for spinal pain were greater for patients with TMD than for controls ($136 vs. $81, 4.8 days vs. 2.7 days, 5.5 days vs. 3.3 days). Higher TMD grade was associated with greater differences in average medical expenditure, number of visits, and lengths of treatment for spinal pain between cases and controls. Additionally, for women, living in a rural area and having an older age and more severe TMD were associated with a greater probability of spinal pain and higher medical expenditure related to spinal pain.

**Conclusion:**

A strong association was observed between the presence of TMD and the presence of spinal pain. The association became stronger as the severity of TMD increased, indicating a positive correlation between the severity of TMD and spinal pain.

## Background

Temporomandibular joint disorder (TMD) is a disease in which structural and functional disorders occur in the temporomandibular joints and related structures [1]. Major clinical symptoms include pain, a clicking sound, and limited range of motion in the jaw joint [2]. According to a cohort study of TMD conducted in the 2019 Orofacial Pain: Prospective Evaluation and Risk Assessment (OPPERA) project, approximately 5–12% of the adult US population experiences painful TMD [3]. According to the 2018 Korea National Health Insurance Service analysis of health insurance claim data, the number of patients with TMD increased by more than 62.8% over 9 years, from 244,708 in 2010 to 398,401 in 2018. In the same period, the medical costs increased by 2.6-fold [4].

TMD is often accompanied by headaches, back pain, joint pain, and abdominal pain [5, 6]. Plesh et al. [7] reported that only 0.77% of patients with TMD did not complain of other associated pain. The management of associated disease is a very important factor in TMD management, because associated disease is related to a poor prognosis [8]. One explanation of the accompanying pain, especially spinal pain in TMD patients, is whole-body imbalance. Dysfunction in TMD can affect factors such as postural asymmetry, center of foot pressure, and spine curvature, which could induce spinal pain [9–12].

Spinal pain is a common and socially important musculoskeletal condition. In the United States, the lifetime prevalence of low back pain is 65 to 80% [13]. In addition, spinal pain is associated with productivity loss, increased medical expenditure, and long-term opioid use [14–16].

Several studies have investigated the relationship between TMD and spinal pain. Wiesinger reported that the incidence of spinal pain increased with the frequency and intensity of temporomandibular joint pain [17]. In a US study, 54% of TMD patients reported neck pain and 64% reported low back pain [7]. Other studies have shown that chronic spinal pain is associated with TMD [18, 19]. Storm [20] showed a significant association between TMD treatment with reduction of cervical spine pain and mobility improvement.

However, the evidence of a correlation between TMD and spinal pain has some limitations: first, a number of these previous studies were performed without a control group [7, 19]; second, when control groups were used, the sample sizes were small, homogeneity between cases and controls was not confirmed, or the analysis was simple, such as a frequency analysis suggesting that TMD was related to spinal pain [17, 18, 21, 22]. Thus, in this study, we used nationwide data from the Health Insurance Review and Assessment (HIRA) database, a representative administrative database of Korea, and propensity score matching (PSM) to overcome limitations of previous studies.

Clarifying the correlation between TMD and spinal pain is important, as secondary prevention can be incorporated into the management and treatment guidelines of TMD if the possibility of spinal pain is known [23]. Therefore, this study aimed to generate a more objective rationale to support the hypothesis that TMD induces spinal pain. The purpose of this study was to identify the prevalence and treatment status of TMD, to analyze the relationship between TMD and spinal pain, and to identify factors affecting spinal pain in patients with TMD. The purpose of this study was to identify the prevalence and treatment status of TMD, to analyze the relationship between TMD and spinal pain, and to identify factors affecting spinal pain in patients with TMD.

## Methods

### Data source

The original data were obtained from the 2016 Korean Health Insurance Review and Assessment Service (HIRA) National Patient Sample. Korea uses a single health insurance system, such that 98% of the population is enrolled in National Health Insurance (NHI) [24]. Claims data in the HIRA are recorded when a claim is made to the corporation for reimbursement of medical services provided by a health care provider. The National Patient Sample data are inclusive of all claims data and randomly stratified according to sex and age, representing the entire Korean population. The National Patient Sample thus comprises cross-sectional data, released yearly, with a sample number of approximately 1.4 million, representing 3% of South Korea’s total population [24].

### Study design and population

This study was a cross-sectional, retrospective study of patients who used medical services at least once in Korea, from January 2016 to December 2016, who were diagnosed with TMD. There have been few studies using International Statistical Classification of Disease (ICD) codes in administrative data. Here, a TMD case was operationally defined as a patient who was diagnosed according to the ICD-10 code K07.6 (temporomandibular joint disorders) or S03.4 (sprain and strain of the jaw), based on internal discussions among research team members.

TMD severity was defined based on the amount of medical services used in relation to the jaw joint. As previous studies have reported that 6 treatments are generally effective [25, 26], we classified the use of fewer than 6 outpatient services as grade 1, 6 or more services but fewer than 12 services as grade 2, and 12 or more outpatient services or inpatient service as grade 3, in terms of TMD severity.

### Propensity score matching

**Fig. 1:**
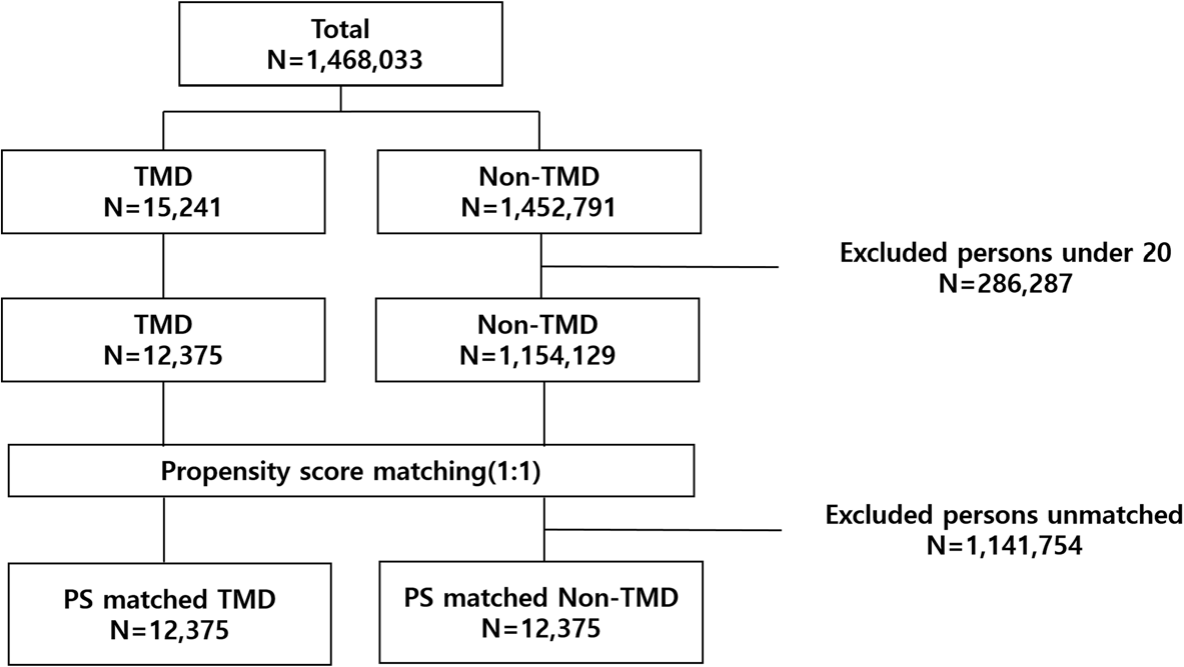
Schematic of the study design. TMD, Temporomandibular Disorder; PS, Propensity Score

In this study, the propensity score-matching method was used to select the control group [27]. The propensity score was calculated according to sex, age, insurance type, region, and Charlson Comorbidity Index (CCI); then, 1:1 matching was performed. Age was classified into 10-year age groups. The regions were classified into Seoul, capital area, metropolitan area, and other areas [28]. Types of insurance were classified as NHI and others (Medicaid); in Korea, Medicaid is a type of health insurance funded by the federal and local governments that provides health coverage for people with low incomes [29]. CCI was initially designed to predict mortality and has been widely used for researchers to measure the burden of disease [30, 31]. CCI was calculated using ICD-10 and the presence of comorbidity was based on whether the code was diagnosed during a 1-year period [32]. Considering the CCI distribution and prior research [33, 34], CCI was classified into 4 grades (0, 1–2, 3–4, and 5 and more). There were 12,375 cases and 1,154,129 controls. After all cases were matched, the final analysis included 12,375 cases and 12,375 controls (Fig. 1).

### Outcomes and other variables

The first outcome was the presence of spinal pain. This was operationally defined as when a patient used medical services due to spinal pain more than once, during a 1-year period. Based on the code applied in previous studies of spinal pain using administrative data [35, 36], and in consultation with 3 specialists in the field of musculoskeletal disease, spinal pain was defined as a diagnosis of ICD-10 M40* (kyphosis and lordosis), M41* (scoliosis), M43* (other deforming dorsopathies), M50* (cervical disc disorders), M51* (other intervertebral disc disorders), M53* (other dorsopathies), M54* (dorsalgia), M99* (biomechanical lesions), S335* (sprain and strain of the lumbar spine), or S134 (sprain and strain of the cervical spine). Spinal pain was also classified into 3 grades and the same criteria were applied as for TMD classification, to ensure consistency [25, 26]. Other variables that indicated the severity of spinal pain included the annual total medical expenses, total numbers of visits, and lengths of treatment due to personal spinal pain, as outcome variables.

The variables related to the use of medical care for patients with TMD were surgery, hospitalization, medical institution type, and medical specialty. Surgery and hospitalization were classified into yes (when TMD surgery and hospitalization were utilized once or more) or no. Medical institution type included general hospital, hospital, and clinic. In Korea, according to the Medical Law, secondary medical institutions with more than 100 beds, 7 or 9 medical specialties, and specialists dedicated to each medical field are called general hospitals. Medical institution type and medical specialty were counted according to the most-used institution type and medical specialty for TMD; thus, counts were not duplicated.

### Statistical analysis

Distributions of categorical sociodemographic variables (sex, age, insurance type, region, CCI, and region) between cases and controls before matching were compared using standardized differences. In this study, because the control group was matched by using a propensity score, the sample were paired. There is no consensus method for comparing variables after propensity score matching, but as the standardized difference is an accepted method according to some previous studies [27, 37] we used it to analyze samples after matching. Standardized difference was also used to analyze samples before matching to compare analysis results between before and after matching groups. The odds ratio for TMD was also calculated to identify risk factors (sex, age, insurance type, region, CCI) for TMD. Distributions of categorical sociodemographic variables between cases and controls after matching were compared using the standardized differences [37, 38]. All categorical variables were summarized as counts and percentages. The prevalence of spinal pain was compared between cases and matched controls for each grade of TMD with the McNemar test because paired categorical samples were used in this study. The difference among TMD grades was also examined using the chi-square test. The prevalence of each grade of spinal pain was compared between cases and matched controls for each grade of TMD with the McNemar–Bowker test, which is used when outcomes are classified into more than 2 categories [39]. Differences among TMD grades and spinal pain grades were also examined using the chi-square test. Medical expenditures, numbers of visits, and lengths of treatment caused by spinal pain were compared between cases and matched controls for each grade of TMD with the paired *t*-test because paired continuous samples were used in this study. A Kolmogorov–Smirnov test revealed the data were not normally distributed, and thus the differences in medical expenditures, numbers of visits, and lengths of treatment among TMD grades were examined using the Kruskal–Wallis test. For post-hoc analysis, Dunnett’s test was performed because the assumption of equal variance was rejected in Levene’s test [40].

For the logistic regression and linear regression analyses, 3 models were constructed by classifying the factors that affect medical use according to characteristics based on previous studies [41, 42]. The model used in the analysis is shown in Eq. (1-1 to 3-2 below.

Model 1: sex, age.

1-1 $$ {Y}_{1i}={\alpha}_1+{\beta}_{11}{X}_{1i}+{\beta}_{12}{X}_{2i}+{\varepsilon}_{1i} $$

1-2 $$ \mathit{\ln}\ {Y}_{2i}={\alpha}_2+{\beta}_{21}{X}_{1i}+{\beta}_{22}{X}_{2i}+{\varepsilon}_{2i} $$

Model 2: Model 1 + (insurance type, region, medical institution type).

2-1 $$ {Y}_{1i}={\alpha}_3+{\sum}_{k=1}^5{\beta}_{3k}{X}_{ki}+{\varepsilon}_{3i} $$

2-2 $$ \ln\ {Y}_{2i}={\alpha}_4+{\sum}_{k=1}^5{\beta}_{4k}{X}_{ki}+{\varepsilon}_{4i} $$

Model 3: Model 2 + (CCI, TMD grade, surgery, hospitalization).

3-1 $$ {Y}_{1i}={\alpha}_5+{\sum}_{k=1}^9{\beta}_{5k}{X}_{ki}+{\varepsilon}_{5i} $$

3-2 $$ \ln\ {Y}_{2i}={\alpha}_6+{\sum}_{k=1}^9{\beta}_{6k}{X}_{ki}+{\varepsilon}_{6i} $$

*Y*_1*i*_:dummy variable with 1 for spinal pain and 0 for without spinal pain in ‘i’th TMD patients.

*Y*_2*i*_: medical expenditure for spinal pain in ‘i’th TMD patients.

*X*_1_: sex, *X*_2_: age, *X*_3_: insurance type, *X*_4_: region, *X*_5_: medical institution type, *X*_6_: CCI, *X*_7_: TMD grade, *X*_8_: surgery, *X*_10_: hospitalization.

Logistic regression analyses were performed for each model to determine the odds ratio of covariates that influence the prevalence of spinal pain in patients with TMD. Linear regression analyses were performed for each model to examine how covariates influence medical expenditure for spinal pain in patients with TMD. Medical expenditure was log-transformed because its distribution was non-normal and shifted to the left [43]. In the logistic regression and linear regression models, sociodemographic variables of the study population and the TMD grade were also included. All analyses were performed with SAS statistical software (version 9.4 for Windows; SAS Institute, Inc., Cary, NC, USA). The significance level was set to 0.05.

## Results

### Sociodemographic characteristics of the study population

Table 1 shows the sociodemographic characteristics of the study population. In 2016, 12,375 patients used medical services more than once due to TMD, which constituted 1.07% of the total study population over 20 years of age. Women were more likely to be affected than men (OR 1.51). Regarding age, most TMD patients were aged 20–29 years and the incidence tended to decrease with age. In addition, possession of NHI, residence in Seoul, and high CCI were risk factors for TMD. After 1:1 propensity score matching, the distributions of all sociodemographic covariates were similar between the two groups (Table 2). While standardized differences were 0.078 to 0.388 before matching, all of them were 0 after matching.

**Table 1:** Sociodemographic Characteristics of Study Population before Matching

	TMD		Non-TMD		Odds Ratio	Standardized difference
	Patients (N)	(%)	Patients (N)	(%)		
Total	12,375	1.1^a^	1,154,129	98.9^a^		
Sex						
Male	4792	38.7^b^	556,880	48.3^b^		
Female	7583	61.3	597,249	51.8	1.51 [1.45, 1.56]	−0.193
Age						
20–29	3958	32.0	181,742	15.7		
30–39	2386	19.3	210,892	18.3	0.52 [0.49, 0.54]	
40–49	2027	16.4	241,739	20.9	0.34 [0.32, 0.37]	
50–59	1879	15.2	237,232	20.6	0.31 [0.29, 0.34]	
60–69	1155	9.3	149,392	12.9	0.28 [,0.25, 0.31]	
70 or older	970	7.8	133,132	11.5	0.24 [0.21, 0.27]	0.388
Insurance type						
NHI	12,073	97.6	1,117,610	96.8		
Others^c^	302	2.4	36,519	3.2	0.88 [0.79, 0.99]	0.298
Region						
Seoul	3116	25.2	252,306	21.9		
Capital area	2712	21.9	260,582	22.6	0.87 [0.83, 0.92]	
Metropolitan	3323	26.9	297,718	25.8	0.94 [0.89, 0.99]	
Rural areas	3224	26.1	343,523	29.8	0.84 [0.80, 0.88]	0.078
CCI						
0	5598	45.2	356,928	30.9		
1–2	4023	32.5	444,973	38.6	1.14 [1.07, 1.22]	
3–4	1971	15.9	258,926	22.4	1.26 [1.13, 1.41]	
5 or more	783	6.3	93,302	8.1	1.53 [1.34, 1.75]	0.298

^a^Denominator: adults who are aged 20 years or older, *N* = 1,166,504

^b^Denominator: total patients by each group, applies to all % below

^c^Others: beneficiaries, veterans

*TMD* Temporomandibular Disorder; *NHI* Nathional Health Insurance, *CCI* Charlson Comorbidity Index

**Table 2:** Sociodemographic Characteristics of Study Population after Propensity Score Matching

	TMD		Non-TMD		
	Patients (N)	(%)	Patients (N)	(%)	Standardized differences
Total	12,375		12,375		
Sex					
Male	4792	38.7^a^	4792	38.7^a^	
Female	7583	61.3	7583	61.3	0.000
Age					
20–29	3958	32.0	3958	32.0	
30–39	2386	19.3	2386	19.3	
40–49	2027	16.4	2027	16.4	
50–59	1879	15.2	1879	15.2	
60–69	1155	9.3	1155	9.3	
70 or older	970	7.8	970	7.8	0.000
Insurance type					
NHI	12,073	97.6	12,073	97.6	
others^b^	302	2.4	302	2.4	0.000
Region					
Seoul	3116	25.2	3116	25.2	
Capital area	2712	21.9	2712	21.9	
Metropolitan	3323	26.9	3323	26.9	
Rural areas	3224	26.1	3224	26.1	0.000
CCI					
0	5598	45.2	5598	45.2	
1–2	4023	32.5	4023	32.5	
3–4	1971	15.9	1971	15.9	
5 or more	783	6.3	783	6.3	0.000

^a^Denominator: total patients by each group, applies to all % below

^b^Others: beneficiaries, veterans

*TMD* Temporomandibular Disorder, *NHI* National Health Insurance, *CCI* Charlson Comorbidity Index

### Prevalence of spinal pain in cases and controls, according to TMD grade

Table 3 shows the prevalence of spinal pain in cases and controls, according to TMD grade. The prevalence of spinal pain was significantly higher in the case group than in the control group for all TMD grades. In addition, when compared by grade, higher TMD grade was associated with an increased prevalence of spinal pain. Changes in prevalence of spinal pain according to TMD grade are shown visually in Additional file 1: Figure S1.

**Table 3:** Prevalence of Spinal Pain of Cases and Controls According to TMD Grade

	Total	Non-spinal pain		Spinal pain		*p*-value^b^
		N	%^a^	N	%^a^	
TMD total						
Control	12,375	8148	65.8	4227	34.2	
Case	12,375	6411	51.8	5964	48.2	<.0001
TMD grade 1^c^						
Control	10,951	7224	66.0	3727	34.0	
Case	10,951	5828	53.2	5123	46.8	<.0001
TMD grade 2^d^						
Control	910	589	64.7	321	35.3	
Case	910	392	43.1	518	56.9	<.0001
TMD grade 3^e^						
Control	514	335	65.2	179	34.8	
Case	514	191	37.2	323	62.8	<.0001
Difference test between TMD grades^f^						<.0001

^a^Denominator: Total TMD patients number or total case/control number by each grade

^b^Analysis of difference in spinal pain frequency according to TMD retention by using McNemar test

^c^TMD grade 1: Outpatient days due to TMD, fewer than 6

^d^TMD grade 2: Outpatient days due to TMD, more than 6 and fewer than 12

^e^TMD grade 3: Outpatient days due to TMD, more than 12 or use of hospitalization services

^f^Analysis of difference in spinal pain frequency between each TMD grades by using Chi-square test

*TMD* Temporomandibular Disorder

### Prevalence of each grade of spinal pain in cases and controls, according to TMD grade

Table 4 shows the prevalence of spinal pain for each grade in the cases and controls, according to TMD grade. The prevalence of spinal pain was higher in cases than in controls for all grades of spinal pain. A higher grade of spinal pain was associated with a greater difference in the prevalence of spinal pain between case and control groups. This suggests that the severity of spinal pain increased with the severity of TMD. There was no trend for the control group in terms of spinal pain level. In all cases, the prevalence of spinal pain was higher in the case group than in the control group. Changes in the prevalence of spinal pain for each level according to TMD grade are shown visually in Additional file 2: Figure S2.

**Table 4:** Prevalence of Spinal Pain for Each Level in Cases and Controls According to TMD Grade

	Total	Spinal grade 1^a^		Spinal grade 2^b^		Spinal grade 3^c^		*p*-value^e^
		N	%^d^	N	%^d^	N	%^d^	
TMD total								
Control	12,375	2806	22.7	614	5.0	807	6.5	
Case	12,375	3525	28.5	1041	8.4	1398	11.3	<.0001
TMD grade 1^f^								
Control	10,951	2489	22.7	541	4.9	697	6.4	
Case	10,951	3155	28.8	884	8.1	1084	9.9	<.0001
TMD grade 2^g^								
Control	910	210	23.1	39	4.3	72	7.9	
Case	910	240	26.4	107	11.8	171	18.8	<.0001
TMD grade 3^h^								
Control	514	107	20.8	34	6.6	38	7.4	
Case	514	130	25.3	50	9.7	143	27.8	<.0001
Difference test between TMD grades^i^								<.0001

^a^Spinal pain grade 1: Outpatient days due to spinal pain, fewer than 6

^b^Spinal pain grade 2: Outpatient days due to spinal pain, more than 6 and fewer than 12

^c^Spinal pain grade 3: Outpatient days due to spinal pain, more than 12 or use of hospitalization services

^d^Denominator: total TMD patients number or total case/control number by each grade

^e^Analysis of difference in spinal pain frequency according to TMD retention by using McNemar-Bowker test

^f^TMD grade 1: Outpatient days due to TMD, fewer than 6 days

^g^TMD grade 2: Outpatient days due to TMD more than 6 and fewer than 12

^h^TMD grade 3: Outpatient days due to TMD more than 12 or use of hospitalization services

^i^Analysis of difference in each spinal pain grade frequency between each TMD grades by using Chi-square test

*TMD* Temporomandibular Disorder

### Medical expenditure, number of visits, and length of treatment for spinal pain in cases and controls, according to TMD grade

Table 5 shows the medical expenditures, numbers of visits, and lengths of treatment for spinal pain in cases and controls, according to TMD grade. For all TMD patients, the medical expenditure for spinal pain in the case group was significantly higher than the expenditure in the control group. Increased TMD grade was associated with increased medical expenditure for spinal pain in both cases and controls; moreover, the difference between the two groups in medical expenses for spinal pain increased with increasing TMD grade. The same tendency was observed for the mean numbers of visits and lengths of treatment. ANOVA showed significant differences between controls and cases by TMD grade with respect to all outcomes (medical expenditures, numbers of visits, and numbers of treatments). In post- hoc analysis with the Dunnett test, differences were significant among all grade groups for all outcomes, except for the difference in numbers of treatment between grades 2 and 3 (Additional file 3: Table S1).

**Table 5:** Medical Expenditure, Number of Visits, and Length of Treatment for Spinal Pain in Cases and Controls by TMD grade

	Average		t Value^a^	Pr > |t|
	Control	Case		
Medical expenditure ($)^b^				
TMD total	81	136	10.34	<.0001
TMD grade 1^c^	78	122	8.62	<.0001
TMD grade 2^d^	96	201	4.61	<.0001
TMD grade 3^e^	106	320	3.77	0.0002
Difference test between TMD grades^g^			95.81	<.0001
Number of visits (days)^f^				
TMD total	2.7	4.8	14.21	<.0001
TMD grade 1^c^	2.6	4.2	11.25	<.0001
TMD grade 2^d^	3.5	7.8	6.50	<.0001
TMD grade 3^e^	3.5	11.3	6.29	<.0001
Difference test between TMD grades			97.51	<.0001
Lengths of treatment (days)^g^				
TMD total	3.3	5.5	10.95	<.0001
TMD grade 1^c^	3.1	4.9	8.95	<.0001
TMD grade 2^d^	3.7	8.6	6.47	<.0001
TMD grade 3^e^	5.3	12.3	3.48	0.0005
Difference test between TMD grades			96.82	<.0001

^a^Results of paired t test

^b^Converted costs according to the U.S. Dollar in 2018.10.12 (US $1.00 = Korean 1130 Won)

^c^TMD grade 1: Outpatient days due to TMD fewer than 6

^d^TMD grade 2: Outpatient days due to TMD more than 6 and fewer than 12

^e^TMD grade 3: Outpatient days due to TMD more than 12 or use of hospitalization services

^f^Number of visits: Number of outpatient visits or number of inpatient care days of patients

^g^Lengths of treatment: Total days of treatment, including drug prescription days without medical treatment

^h^Outcome difference test between TMD grades by using Kruskal–Wallis test

*TMD* Temporomandibular Disorder

### Covariates that influence the prevalence of spinal pain in patients with TMD

Covariates that influenced the prevalence of spinal pain in TMD patients are shown in Table 6; the results of Model 1, Model 2, Model 3 are presented. Among the 3 models, Model 3 had the highest explanatory power. According to Model 3, the probability of spinal pain in patients with TMD was higher in women than in men; it was also higher in older individuals, in those without NHI, and in those living in rural areas (rather than in urban areas) (Table 6). In addition, increased CCI (i.e., increased severity), increased TMD grade, and hospitalization were associated with an increased probability of spinal pain.

**Table 6:** Covariates that Influence Prevalence of Spinal Pain in TMD Patients^a^

	Model 1^b^			Model 2^c^			Model 3^d^		
	Odds	SE	Pr > |t|	Odds	SE	Pr > |t|	Odds	SE	Pr > |t|
Sex (Ref = Male)									
Female	1.21	0.05	0.000	1.22	0.05	0.000	1.19	0.05	0.000
Age (ref = 20–29)									
30–39	1.64	0.09	0.000	1.63	0.09	0.000	1.61	0.09	0.000
40–49	2.17	0.12	0.000	2.11	0.12	0.000	1.48	0.12	0.000
50–59	3.10	0.18	0.000	3.01	0.18	0.000	1.88	0.17	0.000
60–69	4.20	0.30	0.000	4.08	0.29	0.000	2.03	0.25	0.000
70 or older	6.30	0.52	0.000	5.94	0.49	0.000	2.64	0.37	0.000
Insurance type (ref = NHI)									
Others^e^				1.37	0.18	0.015	1.32	0.17	0.033
Region (ref = Seoul)									
Capital area				1.03	0.06	0.627	1.04	0.06	0.467
Metropolitan				1.09	0.06	0.095	1.09	0.06	0.103
Rural areas				1.22	0.06	0.000	1.23	0.07	0.000
Medical institution type (ref = general hospital)									
Hospital				1.52	0.14	0.000	1.53	0.15	0.000
Clinic				2.15	0.18	0.000	2.21	0.18	0.000
CCI (ref = 0)									
2 or less							1.53	0.11	0.000
3–4							2.04	0.23	0.000
5 or more							2.58	0.38	0.000
TMD grade (ref = 1)^f^									
2							1.42	0.11	0.000
3							1.67	0.17	0.000
Surgery (ref = no)									
Yes							0.83	0.10	0.139
Hospitalization (ref = no)									
Yes							2.02	0.80	0.074
AUC			0.659			0.670			0.678

^a^Logistic regression analysis of spinal pain

^b^Model1: sex, age

^c^Model2: Model1 + insurance type, region, medical institution type

^d^Model3: Model2 + CCI, TMD grade, surgery, hospitalization

^e^Others: beneficiaries, veterans

^f^TMD grade 1, Outpatient days due to TMD, fewer than 6; TMD grade 2, Outpatient days due to TMD, more than 6 and fewer than 12; TMD grade 3, Outpatient days due to TMD, more than 12 or use of hospitalization services

*TMD* Temporomandibular Disorder, *NHI* National Health Insurance, *CCI* Charlson Comorbidity Index, *OR* Odds Ratio, *CI* Confidence Interval, *AUC* Area Under the Curve

### Medical expenditure for spinal pain in patients with TMD

Analysis of factors influencing medical expenses resulting from spinal pain in patients with TMD are show in Table 7. Among the 3 models, Model 3 had the highest R square value. The results showed that medical expenses related to spinal pain were higher in women than in men, and were higher in patients with older age than in those with younger age. If the type of insurance was not NHI, the medical expenditure was high; moreover, by region, Seoul (capital city) had the lowest medical expenditure, while rural areas had the highest expenditure. Increased CCI and increased TMD grade were associated with increased expenditure related to spinal pain. The expenditure for TMD was high when the patient was hospitalized for TMD, as well as for patients who used mainly medical clinics rather than general hospitals.

**Table 7:** Medical Expenditure on Spinal Pain in TMD Patients^a^

	Model 1^b^			Model 2^c^			Model 3^d^		
	Estimates	SE	Pr > |t|	Estimates	SE	Pr > |t|	Estimates	SE	Pr > |t|
Sex (Ref = Male)									
Female	0.59	0.10	0.000	0.60	0.10	0.000	0.54	0.10	0.000
Age (ref = 20–29)									
30–39	1.43	0.15	0.000	1.40	0.15	0.000	1.35	0.14	0.000
40–49	2.28	0.15	0.000	2.18	0.15	0.000	1.13	0.22	0.000
50–59	3.44	0.16	0.000	3.33	0.16	0.000	1.91	0.24	0.000
60–69	4.43	0.19	0.000	4.31	0.19	0.000	2.13	0.33	0.000
70 or older	5.69	0.20	0.000	5.46	0.20	0.000	2.9	0.37	0.000
Insurance type (ref = NHI)									
Others^e^				0.96	0.33	0.003	0.85	0.33	0.010
Region (ref = Seoul)									
Capital area				0.06	0.15	0.687	0.1	0.15	0.499
Metropolitan				0.24	0.14	0.084	0.24	0.14	0.091
Rural areas				0.57	0.14	0.000	0.58	0.14	0.000
Medical institution type (ref = general hospital)									
Hospital				1.13	0.24	0.000	1.11	0.24	0.000
Clinic				1.97	0.21	0.000	2.04	0.21	0.000
CCI (ref = 0)									
2 or less							1.22	0.19	0.000
3–4							2.16	0.30	0.000
5 or more							2.96	0.38	0.000
TMD grade (ref = 1)^f^									
2							1.06	0.19	0.000
3							1.57	0.26	0.000
Surgery (ref = no)									
Yes							−0.63	0.32	0.053
Hospitalization (ref = no)									
Yes							2.31	0.93	0.013
R square			0.097			0.108			0.118

^a^Linear regression analysis on medical expenditure due to spinal pain

^b^Model1: sex, age

^c^Model2: Model1 + insurance type, region, medical institution type

^d^Model3: Model2 + CCI, TMD grade, surgery, hospitalization

^e^Others: beneficiaries, veterans

^f^TMD grade 1, Outpatient days due to TMD, fewer than 6; TMD grade 2, Outpatient days due to TMD, more than 6 and fewer than 12; TMD grade 3, Outpatient days due to TMD, more than 12 or use of hospitalization services

*TMD* Temporomandibular Disorder, *NHI* National Health Insurance, *CCI* Charlson Comorbidity Index, *OR* Odds Ratio, *CI* Confidence Interval

## Discussion

The annual period prevalence of TMD in this study was 1.1%, which was relatively low compared to the findings of previous studies (5–40%) [3, 44–46]. This is potentially because TMD was defined only when medical service use occurred due to TMD symptoms, which differed from the approach used in previous studies [25, 44–46]. Assuming a TMD prevalence of 5–40% [3, 44–46] according to studies, 2.8% (1.1/40) to 22% (1.1/5) of TMD patients may use medical services for treatment.

In addition, in the present study, the prevalence of TMD was higher in women and in younger age groups, as in previous studies [6, 45–47]. Some studies have suggested that hormones play a role in the onset of TMD [45, 46]. However, further research is needed regarding the high prevalence of TMD in women. In addition to sex and age, health insurance types and areas of residence were risk factors for TMD. The prevalence of TMD was higher in patients with NHI. There is a medical care system in Korea that guarantees medical assistance for low-income groups [48], and beneficiaries receive assistance without the requirement to enroll in a NHI scheme. Thus, it is implied that patients without NHI may be part of the low-income group. No previous study has investigated the relationship between income and prevalence of TMD, but the results of this study suggest that TMD prevalence is low when income is low. Further study is needed to reveal correlation.

In this study, we found that there was a positive correlation between TMD and spinal pain. Patients with TMD were more likely to have spinal pain than non-TMD patients; moreover, they were more likely to have greater medical expenditure and an increased number of treatments for spinal pain. In addition, a higher degree of TMD was associated with a greater probability of spinal pain; greater severity of spinal pain was associated with increased cost of medical care and increased length of treatment. The medical service use of TMD patients in 2016 Korea (Additional file 4: Table S2) showed that medical expenditure was $1,058,841 per year, which is considerably high. According to this study, although the expenditure due to TMD alone is large, TMD patients with spinal pain have greater expenditure. The high prevalence of spinal pain in patients with TMD may be a result of TMD affecting whole-body posture. Ries and Berzin [9] found that patients with TMD showed greater postural asymmetry than a control group; they concluded that TMD was associated with cervical pain.

A previous study also reported that TMD can affect overall body function; for example, it can influence the location of the center of foot pressure, body sway, and spine curvature [11, 12]. Additionally, the origin of spinal pain in TMD patients may be related to dysregulation of the autonomic nervous system and dysfunction of the hypothalamic–pituitary–adrenal axis [49]. From this perspective, temporomandibular joint pain is a referred pain [49, 50]. Furthermore, patients with TMD tend to have many accompanying diseases [7]. Although TMD is a musculoskeletal disorder, psychosocial factors such as life satisfaction and job satisfaction, mood or emotion, acute trauma, rheumatic diseases, and poor health habits are involved in TMD [46, 51, 52]. Taken together, it can be inferred that there are many factors influencing the accompanying pain of patients with TMD. However, such factors have been poorly studied, with the exception of the twin studies of Visscher et al. [5], who concluded that accompanying pain in TMD was more common in women; they also reported that age, education level, and birth state were not related to accompanying pain.

The risk factors for spinal pain in TMD patients analyzed in this study tended to overlap with the risk factors for spinal pain in the general population. Female sex and older age are well-known risk factors for spinal pain [53, 54]. There have been few studies regarding the relationships between region or income and spinal pain; however, some studies have shown that the prevalence of spinal pain is high in rural areas and among individuals at low-income levels [55, 56]. Thus, factors that cause spinal pain in general also comprise risk factors for spinal pain in TMD patients.

This study had the following limitations. First, the data analyzed in this study were cross-sectional data; therefore, the findings of this study do not confirm causality between TMD and spinal pain; notably, they suggest a positive correlation. The findings support the theory that TMD can affect the whole body by deforming body sway or spine curvature [11, 12]. Further studies using cohort data are needed to determine causality.

Second, this study included limited information regarding clinical data because we analyzed the administrative research data. In particular, the degree of TMD severity is not specified in the administrative data; thus, this study attempted to correct those missing data by including the number of medical services used and CCI. In addition, the presence of pain was not specified in the data; thus, diagnosis was used to define spinal pain and select subjects. Although, diagnosis may not accurately reflect spinal pain, the diagnosis in this study was established as close to spinal pain as possible by referring to previous studies [35, 36] and consultations of specialists. Further studies using hospital data to analyze clinical values such as Range Of Motion (ROM) or Numeric Rating Scale (NRS) and analyzing relationship between TMD and other pain like headache or joint pain is needed.

Nonetheless, this study also had the following advantages. First, this study revealed the effects of various factors (i.e., sex, age, type of health insurance, area, CCI, TMD severity, TMD medical service, and type of medical institution) on the prevalence of spinal pain in TMD patients and the medical expenditure related to spinal pain. Second, we have overcome the limitations of previous studies by using large-scale data that can represent Korea and provide a variety of objective parameters, such as medical expenses and treatment details, as well as by using propensity score-matching methodology. The results of this study provide useful information for the management of TMD and treatment of patients with both TMD and spinal pain. In addition, when spine problems do not continue to improve in general spine treatment, TMD could be considered. In practice, this suggests that consultations with several specialists such as a dentist and neurologist are necessary. The availability of a consultation system can help patients receive quality care and reduce the costs associated with TMD. Further studies to quantify the degree of the correlation between TMD and spinal pain, and to confirm causality, are needed.

## Conclusion

A strong association was observed between TMD and spinal pain. The association became stronger as severity of TMD increased, indicating a positive correlation between severity of TMD and spinal pain. This information can aid in the management of TMD and treatment of patients with both TMD and spinal pain.

## Supplementary information

**Additional file 1: Figure S1.** Prevalence of spinal pain in cases and controls according to TMD grade.

**Additional file 2: Figure S2.** Prevalence of spinal pain for each level in cases and controls according to TMD Grade. spinal1, spinal pain grade 1; spinal2, spinal pain grade 2; spinal3, spinal pain grade 3.

**Additional file 3: Table S1.** Medical Service Use of TMD Patients.

**Additional file 4: Table S2.** Post hoc test for outcome differences between TMD grades.

## Data Availability

The datasets generated and analyzed in the current study are available in the HIRA-NPS repository. The study utilized HIRA data, which are third-party data and thus not owned by the authors. The HIRA data are available upon direct request, via email or fax, and submission of the request form and declaration of data use, which are downloadable from the HIRA website [http://opendata.hira.or.kr/op/opc/selectPatDataAplInfoView.do], and upon payment of a data request fee (300,000 KRW per dataset).

## Abbreviations

AUC: Area Under the Curve
CCI: Charlson Comorbidity Index
CI: Confidence Interval
NHI: National Health Insurance
OR: Odds Ratio
TMD: Temporomandibular Disorder
