# Supplementary figures and images for: The relationship between spinal pain and temporomandibular joint disorders in Korea: a nationwide propensity score-matched study
Source: BMC Musculoskelet Disord. 2019 Dec 29;20:631. doi: 10.1186/s12891-019-3003-4 (PMC6935481; doi:10.1186/s12891-019-3003-4)

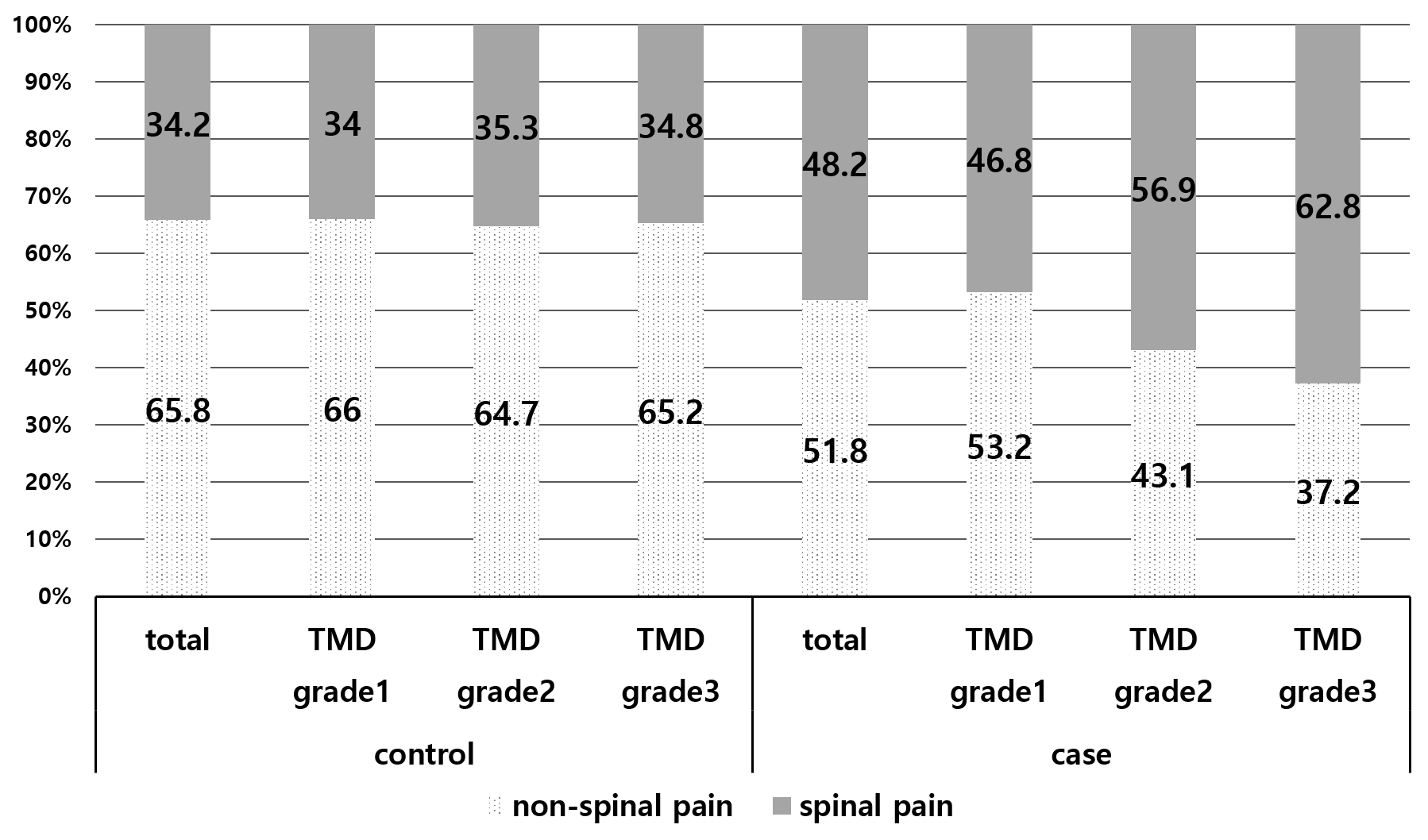

Supplement: Supplementary file 1 — Additional file 1: Figure S1. Prevalence of spinal pain in cases and controls according to TMD grade. [file 12891_2019_3003_MOESM1_ESM.png]

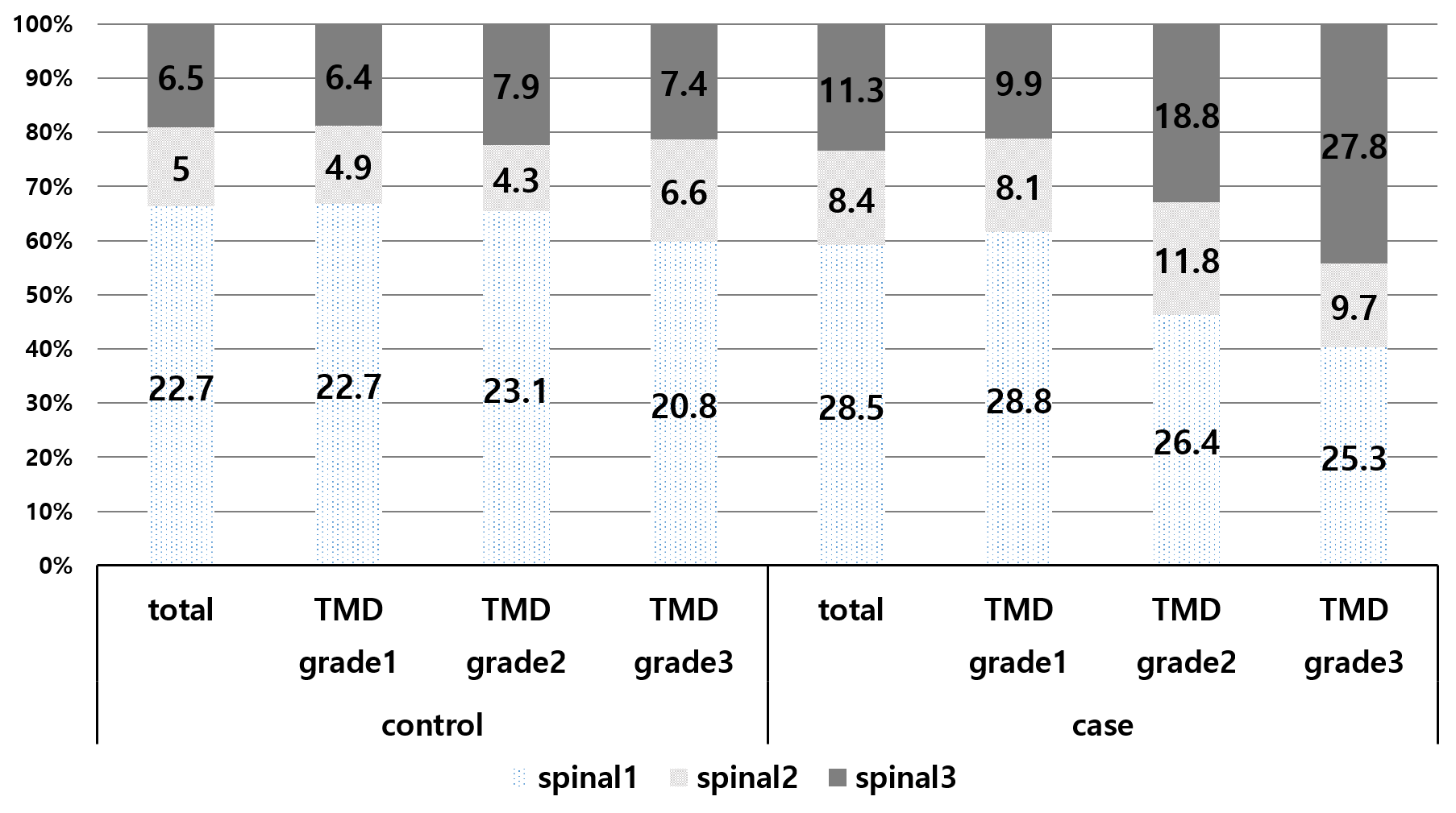

Supplement: Supplementary file 2 — Additional file 2: Figure S2. Prevalence of spinal pain for each level in cases and controls according to TMD Grade. spinal1, spinal pain grade 1; spinal2, spinal pain grade 2; spinal3, spinal pain grade 3. [file 12891_2019_3003_MOESM2_ESM.png]
