# Supplementary material for: The relationship between spinal pain and temporomandibular joint disorders in Korea: a nationwide propensity score-matched study
Source: BMC Musculoskelet Disord. 2019 Dec 29;20:631. doi: 10.1186/s12891-019-3003-4 (PMC6935481; doi:10.1186/s12891-019-3003-4)
Supplement: Supplementary file 3 — Additional file 3: Table S1. Medical Service Use of TMD Patients. [file 12891_2019_3003_MOESM3_ESM.docx]

**Table S1. Medical Service Use of TMD Patients**

|  | **TMD patients** | | **Medical expenditure** | | **Medical expenditure*†** | **Number of visits‡** | **Length of treatment§** |
| --- | --- | --- | --- | --- | --- | --- | --- |
|  | **N** | **%** | **$*** | **%** |  |  |  |
| Total | 12,375 |  | 1,058,841 |  | 86 | 2.8 | 2.9 |
| Surgery |  |  |  |  |  |  |  |
| Yes | 308 | 2.5 | 76,178 | 7.2 | 247 | 5.0 | 5.5 |
| No | 12,067 | 97.5 | 982,663 | 92.8 | 81 | 2.8 | 2.9 |
| Hospitalization |  |  |  |  |  |  |  |
| Yes | 39 | 0.3 | 61,666 | 5.8 | 1,581 | 11.9 | 16.2 |
| No | 12,336 | 99.7 | 997,175 | 94.2 | 81 | 2.8 | 2.9 |
| Medical institution type |  |  |  |  |  |  |  |
| General hospital | 786 | 6.4 | 94,958 | 9.0 | 121 | 2.9 | 3.2 |
| Hospital | 1,667 | 13.5 | 223,680 | 21.1 | 134 | 3.2 | 3.4 |
| Clinic | 9,922 | 80.2 | 740,202 | 69.9 | 75 | 2.8 | 2.8 |
| Medical specialty |  |  |  |  |  |  |  |
| Conservative dentistry | 2,709 | 21.9 | 198,563 | 18.8 | 73 | 2.4 | 2.5 |
| Orthopedics surgery | 1,974 | 16.0 | 124,209 | 11.7 | 63 | 2.4 | 2.5 |
| Oral and maxillofacial surgery | 1,477 | 11.9 | 120,868 | 11.4 | 82 | 2.2 | 2.2 |
| Oral medicine | 1,350 | 10.9 | 211,406 | 20.0 | 157 | 3.9 | 4.0 |
| Acupuncture and moxibustion medicine | 1,014 | 8.2 | 97,780 | 9.2 | 96 | 4.2 | 4.3 |
| Internal Oriental medicine | 1,018 | 8.2 | 107,918 | 10.2 | 106 | 4.6 | 4.9 |
| Internal medicine | 705 | 5.7 | 39,512 | 3.7 | 56 | 2.4 | 2.5 |
| Periodontology | 503 | 4.1 | 30,918 | 2.9 | 61 | 1.4 | 1.5 |
| Neurosurgery | 446 | 3.6 | 46,463 | 4.4 | 104 | 2.0 | 2.2 |
| Otorhinolaryngology | 387 | 3.1 | 12,658 | 1.2 | 33 | 2.0 | 2.0 |
| Anesthesiology | 251 | 2.0 | 16,868 | 1.6 | 67 | 2.4 | 2.4 |
| General Surgery | 157 | 1.3 | 11,267 | 1.1 | 72 | 2.0 | 2.0 |
| Oriental Medicine Rehabilitation | 141 | 1.1 | 25,238 | 2.4 | 179 | 5.3 | 5.8 |
| Ophthalmology | 118 | 1.0 | 9,223 | 0.9 | 78 | 2.9 | 3.0 |
| Family Medicine | 125 | 1.0 | 5,948 | 0.6 | 48 | 1.7 | 1.7 |

*Converted costs according to the exchange rate in 2018.10.12 (US $1.00 = Korean 1,130 Won).
†cost per person ($).
‡Number of visits: Days visited by actual medical institutions, such as hospitalization and outpatient.
§Length of treatment: Number of treatment days, including drug prescription days.
TMD, Temporomandibular Disorder
