# Supplementary material for: The relationship between spinal pain and temporomandibular joint disorders in Korea: a nationwide propensity score-matched study
Source: BMC Musculoskelet Disord. 2019 Dec 29;20:631. doi: 10.1186/s12891-019-3003-4 (PMC6935481; doi:10.1186/s12891-019-3003-4)
Supplement: Supplementary file 4 — Additional file 4: Table S2. Post hoc test for outcome differences between TMD grades. [file 12891_2019_3003_MOESM4_ESM.docx]

**Table S2. Post hoc test for outcome differences between TMD grades.**

| TMJ level | p-value* |
| --- | --- |
| Medical expenditure | |
| 2 vs 1 | 0.0058 |
| 3 vs 1 | <.0001 |
| 2 vs 3 | 0.0015 |
| Number of visits | |
| 2 vs 1 | <.0001 |
| 3 vs 1 | <.0001 |
| 2 vs 3 | 0.0001 |
| Number of treatments | |
| 2 vs 1 | 0.0002 |
| 3 vs 1 | <.0001 |
| 2 vs 3 | 0.1405 |

*Results of Dunnett's test
